# In Vitro Antiproliferative Activity of Fucoidans Extracted From *Sargassum natans* and *Sargassum fluitans* in the HeLa Cell Line

**DOI:** 10.1155/ijfo/3001089

**Published:** 2025-11-14

**Authors:** S. R. Vázquez-Rodríguez, A. Cavazos-Garduño, C. R. Cortez-Álvarez, M. C. del Toro-Castillo, J. C. Serrano-Niño

**Affiliations:** ^1^ Department of Pharmacobiology, University Center of Exact Sciences and Engineering, University of Guadalajara, Guadalajara, Jalisco, Mexico, udg.mx; ^2^ Department of Translational Bioengineering, University Center of Exact Sciences and Engineering, University of Guadalajara, Guadalajara, Jalisco, Mexico, udg.mx

**Keywords:** antioxidant, antiproliferative, cancer, fucoidans, *Sargassum*

## Abstract

This paper presents an analysis of the polyphenol content, antioxidant capacity, and antiproliferative activity of fucoidans extracted from *Sargassum natans* and *Sargassum fluitans*. The results obtained indicated a high total polyphenol content (113 ± 2.3 mg GAE/g), which is probably associated with great antioxidant capacity. Antioxidant activity determined by the DPPH and FRAP methods resulted in 42.21*%* ± 0.864*%* inhibition and 1911 ± 71 mM TE/g, respectively. The degree of sulfation for the extracted fucoidans was found to be, using a turbidimetric method with BaCl_2_, at 13.2% for *S. natans* and 15.6% for *S. fluitans.* In addition, intrinsic viscosity determination allowed molecular weights to be estimated using the Mark–Houwink equation as 26.2 and 18.7 kDa for *S. natans* and *S. fluitans*, respectively. The antiproliferative properties of fucoidans extracted from *Sargassum* were evaluated through the MTT assay on HeLa cervical cancer cells. The study revealed a marked reduction in cell proliferation, most notably at higher concentrations (4000 *μ*g/mL), where the inhibition rate reached 90.80%, with an IC_50_ value of 1277.79 *μ*g/mL. These findings emphasize the strong antiproliferative effects of *Sargassum*‐derived fucoidans, believed to function through pathways such as apoptosis induction, cell cycle modulation, and suppression of cellular growth. The observed biological activity appears strongly tied to structural characteristics like sulfation patterns and molecular weight, which are critical determinants of fucoidan functionality. This aligns with earlier research highlighting the antitumor and antioxidant properties of fucoidans. Moving forward, in vivo studies will be essential to validate their safety profile and therapeutic potential in animal models. Furthermore, detailed mechanistic investigations should be prioritized to uncover the pathways driving their anticancer effects.

## 1. Introduction

*Sargassum* is the common name for two marine species of the genus of brown macroalgae (*Sargassum*), of which 361 species are currently recognized. Of these, two species, *Sargassum natans* and *Sargassum fluitans*, spend their lives floating in the sea [1]. In recent years, large deposits of these algae have significantly affected the coasts of the Mexican Caribbean. This phenomenon, driven by climate change, has environmental, social, and economic repercussions. However, these algae also represent a valuable source of bioactive compounds with antioxidant and antitumor properties. Brown algae contain large amounts of various polysaccharides, including cellulose, fucoidans, and alginic acids. Of these compounds, fucoidans are the most notable from a nutraceutical perspective. Fucoidans are a group of sulfated polysaccharides containing fucose and consist of mixtures of related polysaccharides, with varying amounts of monosaccharide residues and noncarbohydrate components, such as sulfate and acetyl groups [2]. Fucoidans have been shown to possess several biological properties, including antioxidant, anticoagulant, anti‐inflammatory, antiangiogenic, and anticancer properties, as well as neuroprotective effects [3]. Furthermore, fucoidans have been reported to trigger programmed cancer cell death by generating stress in the endoplasmic reticulum, which activates proteins that induce apoptosis. This process can also occur through the mitochondrial pathway when reactive oxygen species (ROS) levels within the cell are elevated. It is also associated with caspase activation and mitochondrial dysfunction, including dissipation of mitochondrial membrane potential, disruption of calcium homeostasis, release of cytochrome c, and reduced expression of antiapoptotic proteins [4, 5]. The extraction of sulfated polysaccharides containing fucose from brown algae generally requires multiple prolonged aqueous extractions, often using hot acid and acids such as hydrochloric or sulfuric acid, sometimes adding calcium chloride to precipitate the alginate. Furthermore, factors such as extraction time, temperature, and acid concentration or pH have been shown to influence both the yield and composition of fucoidan or sulfated polysaccharides containing fucose, as well as their biological activity [6]. The present study was aimed at evaluating for the first time the in vitro antiproliferative activity of fucoidans extracted from *S. natans* and *S. fluitans* on the cervical cancer cell line HeLa, highlighting their scientific relevance as unexplored sources of bioactive polysaccharides and their potential as a sustainable alternative for the valorization of massive *Sargassum* bloom in the Mexican Caribbean.

## 2. Materials and Methods

### 2.1. Sample Collection and Preparation

The brown algae *S. natans* and *S. fluitans* were collected manually at a maximum depth of 1 m off the coast of Playa Delfines (21.007039, −86.773150), Cancun, Quintana Roo, Mexico, in May 2023. By maintaining a single site, season, and standardized postharvest handling, environmental and biological variability that could influence the content of bioactive compounds was reduced. After collection, the algae were washed with abundant running water to remove epiphytes and sand residues and dried at room temperature for 24 h. The dried algae were ground in a blade processor and sieved using 40‐mesh sieves. The ground material was kept refrigerated until use. Species identification was performed using the illustrated guide to pelagic and benthic *Sargassum* species present in the Mexican Caribbean shorelines (SargaZoom). This guide includes descriptions, an image gallery, and ecological information and is the result of work carried out at the Applied Phycology Laboratory, Cinvestav, Mérida, Mexico.

### 2.2. Fucoidan Extraction

Fucoidan extraction was carried out using the methodology proposed by Chen et al. [7], where 10 g of dried algae (both species) were first mixed with 25 mL of deionized water and then sonicated using an ultrasonicator (SFX 550, Branson, Mexico) with a power of 55 W and an amplitude of 60%. The sonicated mixture was heated at 65°C for 1 h and then centrifuged at 3500 rpm for 10 min to separate the supernatant. The supernatant was collected and mixed with CaCl_2_ (1%), and the solution was kept overnight at 4°C to precipitate the alginic acid. Centrifugation was then performed again at 15,000 rpm for 10 min, and the resulting supernatant was collected. This supernatant was mixed with 96% ethanol and kept at 4°C overnight. This solution was then filtered to obtain the fucoidans. The resulting fucoidans were dried in a dehydrator (Hamilton Beach 32100A, China) at 50°C, and the powder was packaged in an airtight container for further characterization.

### 2.3. Yield and Biochemical Composition of Fucoidans

The extraction yield of fucoidans was calculated as the percentage of dry extract obtained relative to the initial dry weight of *Sargassum* samples. Total carbohydrate content was calculated using the phenol–sulfuric acid method with glucose as a standard, and the result was expressed as milligrams of glucose equivalents per gram of dry extract. Protein content was determined by Bradford assay using bovine serum albumin as a standard and expressed as milligrams of protein per gram of dry extract.

### 2.4. Degree of Sulfation of Fucoidans

The degree of sulfation of fucoidans was determined by a turbidimetric method, based on the formation of a barium sulfate (BaSO_4_) precipitate in an acidic medium. The fucoidans were subjected to acid hydrolysis with HCl (1 M) at 100°C for 2 h. Subsequently, the neutralized extract was treated with barium chloride (BaCl_2_) (10%), and the resulting turbidity was measured at 420 nm. A calibration curve with sodium sulfate (Na_2_SO_4_) was used to express the sulfate content in micrograms per milligram. The degree of sulfation was estimated as a percentage of the sulfate content with respect to the total weight of the extract.

### 2.5. Molecular Weight (Mw) Estimation of Fucoidans

The average Mw of fucoidans was estimated by viscometric analysis using an Ostwald viscometer at 25°C. Aqueous solutions of the extract were prepared at different concentrations (0.1–0.5% *w*/*v*), and the flow time was measured compared to distilled water. The relative viscosity was calculated and extrapolated to obtain the intrinsic viscosity. Finally, the Mark–Houwink equation was applied using constants previously reported for fucoidans (*K* = 3.4 × 10^−4^, *a* = 0.72), which gave an estimate of the Mw.

### 2.6. Fourier Transform Infrared (FT‐IR) Characterization of Fucoidans

The obtained fucoidans were analyzed using FT‐IR spectroscopy (Cary 630, Agilent Technologies, United States) to compare the presence of functional group characteristics of fucoidans. Spectra were recorded (4000–450 cm^−1^) at a nominal resolution of 2 cm^−1^ and acquired at 32 scans per sample.

### 2.7. Quantification of Phenolic Compounds and Antioxidants of Fucoidans

To evaluate the antioxidant capacity and total polyphenol content, the obtained fucoidans were reconstituted in distilled water at a concentration of 200 *μ*g/mL. A Trolox (6‐hydroxy‐2,5,7,8‐tetramethyl‐chromane‐2‐carboxylic acid, Sigma‐Aldrich) standard curve was prepared at concentrations of 10–500 *μ*M for antioxidant capacity, while to assess total polyphenol content, a gallic acid (Golden Bell) standard curve was prepared using concentrations of 10–100 *μ*g/mL to calculate gallic acid equivalents.

### 2.8. Detection and Quantification of Total Polyphenols in Fucoidans

Then, 250 *μ*L of reconstituted fucoidans, 1500 *μ*L of distilled water, and 125 *μ*L of Folin–Ciocalteu reagent (F9252, Sigma‐Aldrich, United States) were placed in a tube and allowed to react for 5 min at room temperature. Subsequently, 500 *μ*L of distilled water and 375 *μ*L of 7.5% Na_2_CO_3_ (Golden Bell) were added, the mixture was homogenized, and the reaction was incubated for 2 h in the dark. After this time, 200 *μ*L of the reaction mixture was placed in triplicate in 96‐well microplates, and the absorbance at 750 nm was measured. The reaction mixture and 250 *μ*L of the solvent (double‐distilled water) were used as a blank. A gallic acid standard curve (Golden Bell) was prepared using concentrations of 10–100 *μ*g/mL to calculate gallic acid equivalents.

### 2.9. 2,2‐Diphenyl‐1‐Picrylhydrazyl (DPPH) Radical Scavenging Activity of Fucoidans

Briefly, a solution was prepared by adding 1350 *μ*L of 150 *μ*M DPPH (D9132, Sigma‐Aldrich) in methanol (Golden Bell) and 200 *μ*L of the extract to a microcentrifuge tube and incubating in the dark for 30 min. Subsequently, 200 *μ*L of the mixture was placed in triplicate in a 96‐well plate, and the absorbance at 515 nm was measured in a spectrophotometer (Multiskan Sky, Thermo Scientific) to calculate the percentage of inhibition. Methanol with DPPH was used as the blank and ascorbic acid as a positive control. The percentage of inhibition was calculated using the following formula:

%inhibition=Absblank−AbssampleAbsblank×100.

### 2.10. Determination of Ferric Reducing Antioxidant Power (FRAP) of Fucoidans

The FRAP solution was prepared, and 75 *μ*L of the extract and 1425 *μ*L of the FRAP solution were added to a test tube and incubated in the dark for 30 min at 37°C. In a 96‐well plate, 200 *μ*L of the reaction mixture was added in triplicate, and the absorbance was read at 593 nm. The solvent and the FRAP reaction mixture were used as blanks. The Trolox equivalents of the sample were calculated using a Trolox standard curve (238813, Sigma‐Aldrich) at concentrations ranging from 10 to 500 *μ*M.

### 2.11. Evaluation of Antitumor Activity of Fucoidans

To evaluate antitumor activity, the adherent human cervical adenocarcinoma (HeLa) cell line was used. HeLa cells were purchased from ATCC (CCL‐2, Manassas, VA, United States). Cells were cultured in 33 mm Petri dishes and maintained in complete DMEM (Sigma‐Aldrich) and incubated at 37°C, 5% CO_2_, and 95% relative humidity [8].

### 2.12. Cell Viability Counting

Subcultures were performed from monolayers at 80%–90% confluence. The culture medium was discarded, and 1 mL of trypsin (0.5%) was added, incubating for 1 min. The solution was then transferred using a 1000 *μ*L micropipette to a 1 mL conical vial and centrifuged at 2000 rpm for 2 min. The supernatant was discarded, and the pellet was preserved. Finally, the cells were resuspended in 1 mL of complete culture medium.

Cell viability was counted using 10 *μ*L of trypan blue (0.4%) and 10 *μ*L of cells in suspension. Then, 10 *μ*L of the solution was added in a 1:1 ratio to the Neubauer chamber and viewed under an inverted microscope (Olympus model IX50) following the protocol described by Strober [9]. The concentration was calculated using the following formula:

Concentration CellsmL: Total cells countedSquare numbers×dilution×104.

### 2.13. Antiproliferative Assay of *S. natans* and *S. fluitans* Fucoidans

To evaluate the antiproliferative effect, powdered fucoidans were reconstituted with DMEM (Sigma‐Aldrich). The number of HeLa cells seeded was 5000 cells/well. To allow cell adherence, the cells were incubated for 18 h at 37°C. After 18 h, 100 *μ*L of each of the following treatment groups was added: (a) positive control (sodium dodecyl sulfate 0.029 mg/mL), (b) negative control (DMEM), (c) fucoidans (4000 *μ*g/mL), (d) fucoidans (2000 *μ*g/mL), (e) fucoidans (1000 *μ*g/mL), (f) fucoidans (500 *μ*g/mL), and (g) fucoidans (250 *μ*g/mL). The microplates were incubated for 72 h. The antiproliferative effect was evaluated using the MTT assay as follows: 5000 cells/well were seeded in a sterile 96‐well microplate and incubated for 18 h to ensure adherence. Each of the fucoidan concentrations to be evaluated (250, 500, 1000, 2000, 3000, and 4000 *μ*g/mL) was then added in triplicate. The microplates were incubated for 72 h. Then, 100 *μ*L of MTT (0.5 mg/mL in PBS) was added to each treated well and incubated at 37°C, with 5% CO_2_ and 95% relative humidity for 4 h. Finally, the supernatant was discarded, and 100 *μ*L of dimethyl sulfoxide (DMSO) was added and incubated for 20 min in the dark. The wells with the highest cell viability had a purple color, while the wells with the highest cell death had a yellowish‐transparent color. The percentage of cell viability was evaluated using the MTT assay, applying the following formula:

%cell viability=DE treated cells−DO blankDE control cells−DO blank×100.

Cell viability percentages below 40% are considered strong cytotoxicity, between 60% and 40% are considered moderate cytotoxicity, between 80% and 60% are considered weak cytotoxicity, and percentages of cell viability above 80% are considered as non‐cytotoxicity.

### 2.14. Statistical Analysis

All experiments were performed in triplicate and repeated independently at least three times. Total polyphenol and antioxidant capacity values were reported as the mean and standard deviation. The IC_50_ values were calculated by fitting the cell viability data to a nonlinear regression curve using a sigmoidal dose–response model in GraphPad Prism 9.0 (GraphPad Software, San Diego, CA, United States). This method allows accurate estimation of the concentration of fucoidans required to inhibit 50% of HeLa cell proliferation, providing a robust statistical approach compared to linear interpolation. The statistical analysis to compare the differences in vitro concentrations of antitumor activity tested consisted of a simple analysis of variance using Fisher′s test (*α* = 0.05) using the MINITAB statistical package.

## 3. Results and Discussion

### 3.1. Yield and Biochemical Composition of Fucoidans

The extraction yield of fucoidans was 4.8% (*w*/*w*) for *S. natans* and 5.3% for *S. fluitans*, calculated as the percentage of dry extract obtained relative to the initial algal dry weight. The biochemical composition of the extract showed a total carbohydrate content of 612 ± 15 mg/g and 587 ± 18 mg/g for *S. natans* and *S. fluitans*, respectively, determined by the phenol–sulfuric acid method. Protein content, quantified by the Bradford assay, was 21 ± 2 mg/g for *S. natans* and 19 ± 3 mg/g for *S. fluitans*. These data confirm that the extracts are rich in carbohydrates, as expected for fucoidans with only minor protein content.

### 3.2. Degree of Sulfation and Mw Estimation of Fucoidans

The sulfate groups present in the extracted fucoidans were quantified by the turbidimetric method with BaCl_2_ using a standard curve based on Na_2_SO_4_. The curve showed a direct linear relationship between the sulfate concentration (0–50 *μ*g/mL) and the absorbance measured at 420 nm, with a coefficient of determination *R* ^2^ equal to 0.997. The absorbances of the fucoidan samples analyzed were 0.305 for *S. natans* and 0.361 for *S. fluitans*, resulting in estimated concentrations of 32.6 and 39.8 *μ*g of sulfate per milligram of sample, respectively. Regarding the degree of sulfation, it was 13.2% for *S. natans* and 15.6% for *S. fluitans*, values that are within the range reported in the literature for fucoidans with significant biological activity. These results demonstrate a notable presence of sulfate groups in the extracts, which could play a relevant role in their antioxidant and antiproliferative properties.

### 3.3. FT‐IR Characterization of Fucoidans Extracted From *S. natans* and *S. fluitans*

**Figure 1:**
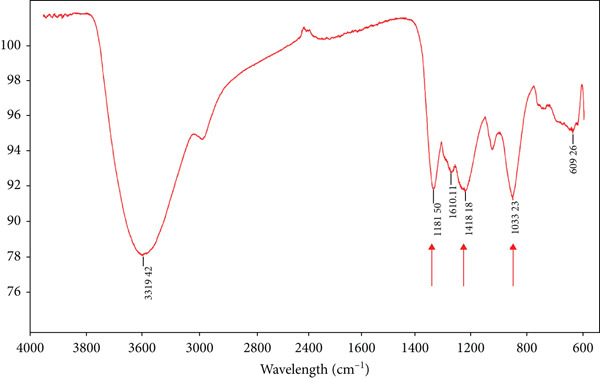
FT‐IR spectrum of fucoidans extracted from *Sargassum natans* and *Sargassum fluitans.*

Figure 1 shows the FT‐IR spectrum of fucoidans extracted from *S. natans* and *S. fluitans*, highlighting the presence of groups such as uronic acids, galactose, fucose, and sulfate groups. FT‐IR spectroscopy analysis confirmed the presence of functional groups characteristic of sulfated polysaccharides in the fucoidan extract obtained from *S. natans* and *S. fluitans*. A weak band near 2931 cm^−1^ was identified in the spectrum, corresponding to the aliphatic C–H stretching vibrations present in the sugar backbones. An absorption signal at 1615 cm^−1^ was associated with angular deformation of the absorbed water or vibrations of the carbonyl group (C=O), while at 1418 cm^−1^, a band attributable to C–H and –OH deformations was recorded. The most relevant band appeared at 1251 cm^−1^, assigned to the asymmetric stretching of the S=O bond, confirming the presence of sulfate groups, characteristic of fucoidan. In the region of 1028 cm^−1^, a band corresponding to the stretching of C–O–C and C–O bonds, common in the glycosidic bonds of polysaccharides, was detected. Finally, a weak band around 609 cm^−1^ could be associated with C–O–S bond deformations or sulfated ring vibrations. These results are consistent with spectral profiles reported in the literature for fucoidan extracted from brown algae, such as *Fucus vesiculosus*, *Stephanocystis dioica*, *Ascophyllum nodosum*, and *Sargassum* species. Overall, the spectral pattern obtained confirms the presence of fucoidan in the analyzed extract, evidenced by the combination of signals attributable to hydroxyl, glycosidic, carboxylic, and sulfated groups [10]. A limitation of the present study is that fucoidan characterization was performed exclusively by FT‐IR spectroscopy; while this technique confirmed the presence of characteristic functional groups, a more complete structural elucidation requires complementary analysis, such as nuclear magnetic resonance (NMR) spectroscopy and monosaccharide composition profiling. These additional approaches will be addressed in future studies to provide a more detailed understanding of the structural features responsible for biological activities.

### 3.4. Quantification of Phenolic Compounds and Antioxidants

**Figure 2:**
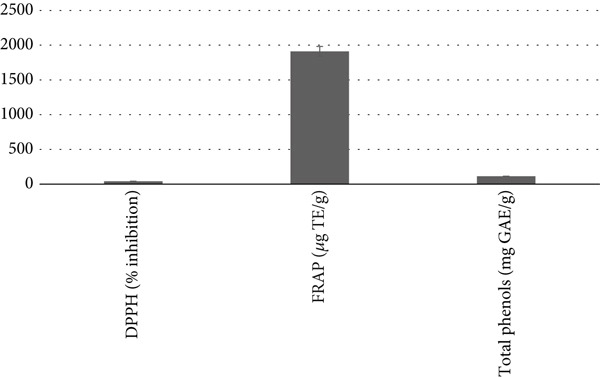
Antioxidant capacity and total polyphenol content of fucoidans extracted from *Sargassum natans* and *Sargassum fluitans*. Values are expressed as mean ± standard deviation (SD). GAE, gallic acid equivalents; TEs, Trolox equivalents; DPPH, 2,2‐diphenyl‐1‐picrylhydrazyl; FRAP, ferric reducing antioxidant power.

Figure 2 shows the antioxidant capacity and total polyphenol content values of fucoidans extracted from *S. natans* and *S. fluitans*. The antioxidant capacity, assessed by both the DPPH and FRAP methods, reached an inhibition of 42.21*%* ± 0.864*%* in the DPPH assay, while the FRAP method yielded 1911 ± 71 * μ*g of Trolox equivalents per gram. The total polyphenol content was 113 ± 2.3 mg of gallic acid equivalents per gram of fucoidan extract. These results are lower than those reported by Palanisamy et al. [11], where fucoidans isolated and purified by DEAE cellulose ion exchange chromatography and dialysis from *Sargassum polycystum* had a DPPH inhibition of 55.94*%* ± 0.69*%*. This may be due to the extraction method, especially the isolation and purification process of fucoidans, which could result in a higher concentration of fucoidans with sulfate groups in the final product. The results obtained in this study showed a remarkably high antioxidant activity compared to those reported by Tsou et al. [12], who carried out extractions at two different temperatures (65°C and 80°C) of *Sargassum ilicifolium* and obtained antioxidant activity percentages of 81% and 78% at concentrations of 1 mg/mL, respectively. It has been reported that the variability between the antioxidant capacity and composition of bioactive compounds could be attributed to factors inherent to the algal species, its collection site, and even the microenvironment where they develop [13]. In the study carried out by Rajauria et al. [14] on the evaluation of the molecular properties of fucoidan extracted from *A. nodosum* using assisted hydrothermal extraction (AEH) (120°C, 62 min), it was observed that the percentage of inhibition of DPPH radicals obtained (43.0%) was very similar to the results obtained in this study, this could be attributed to the extraction methods used, where hydrothermal‐assisted extraction involves the use of water at high temperatures and pressures, which can facilitate the release of fucoidans from the algal matrix, in addition to the fact that high temperatures can favor the decomposition of cellular structures and promote the release of the desired compounds, as in ultrasound‐assisted extraction that uses high‐frequency sound waves to generate vacuum cavities in the extraction medium, facilitating the rupture of cells and the release of fucoidans. Regarding the results obtained for antioxidant capacity using the FRAP test, the crude *Sargassum* extract showed a result of 38.6 ± 0.2 mg/g, while the purified fraction was 30.8 ± 0.4 mg/g. These results are significantly lower than those reported by Rajauria et al. [14], who reported a value of 1911 ± 71 * μ*g TE/g fucoidans obtained from the brown alga *A. nodosum*. The discrepancy between the results can be attributed to a variety of factors that may influence the extraction process and sample conditions. The extraction procedures used in both studies may have differed in terms of parameters such as temperature, extraction time, and solvent type, among others. Furthermore, the harvesting conditions of the algae, as well as natural variations in the chemical composition of the algal samples, may have also contributed to the differences in the results. According to Sinurat and Maulida [15], antioxidant capacity increases in direct relation to the amount of fucoidan sulfate present. Therefore, an increase in sulfate content leads to an increase in antioxidant activity. It is also important to mention how extraction methods can influence the composition and antioxidant activity of fucoidans. It can be argued that, although the extraction method used in this study may be considered simple, it can still provide fucoidans with significant antioxidant activity, highlighting its potential application.

### 3.5. Evaluation of Antitumor Activity

**Figure 3:**
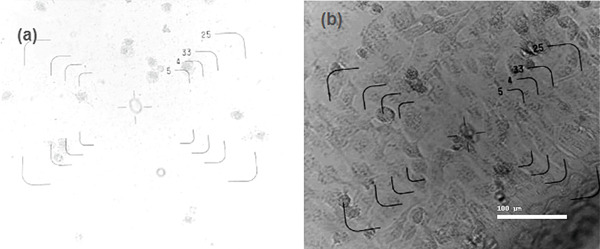
Antitumor activity of fucoidans extracted from *Sargassum natans* and *Sargassum fluitans* in the HeLa cell line. (a) Culture treated with 4000 *μ*g/mL for 72 h. The treated culture showed a reduction in cell density, loss of morphology (rounded cells), and cellular debris. (b) HeLa cells without fucoidan treatment.

**Figure 4:**
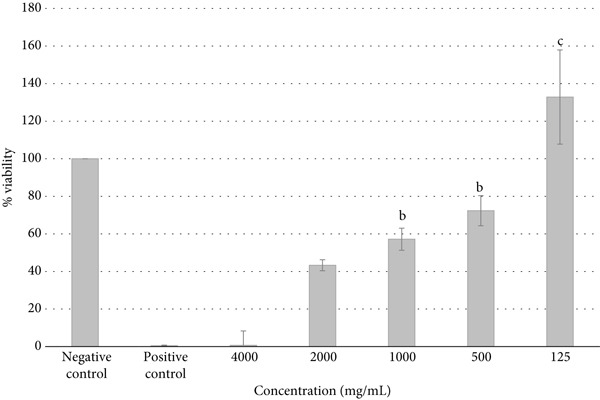
Effect of fucoidans on HeLa cell viability. Cells were treated at concentrations ranging from 125 to 4000 *μ*g/mL of fucoidans for 72 h.

**Figure 5:**
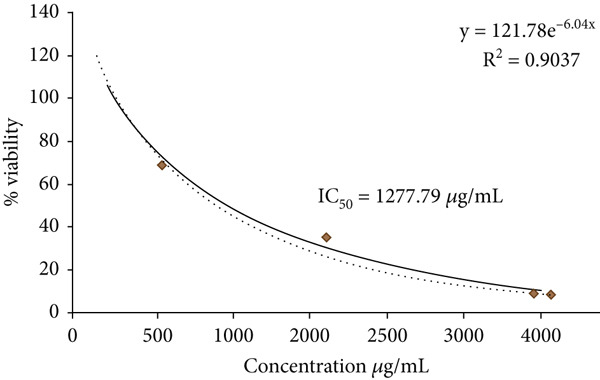
Determination of the IC_50_ of fucoidans on the HeLa cell line. The cultures were treated with different concentrations of fucoidans for 72 h.

Figure 3 shows the antitumor activity of fucoidans extracted from *S. natans* and *S. fluitans* in the HeLa cell line. Treatment with 4000 *μ*g/mL of fucoidans for 72 h (Figure 3a) caused a reduction in cell density, loss of morphology (rounded cells), and the presence of cellular debris. In contrast, HeLa cells without fucoidan treatment remained intact (Figure 3b). It is observed that cells treated with the extract showed a dose‐dependent decrease in the number of viable cells compared to untreated cells, suggesting that the extract possesses antiproliferative activity. At higher concentrations (4000 *μ*g/mL), cell viability barely reached 10% after 24 h of contact with the extract; that is, the extract is capable of being toxic enough to eliminate 90% of the cells to which it is exposed (IC_90_ concentration = 4000 * μ*g/mL). In contrast, at the lowest concentration tested (125 *μ*g/mL), an increase in cell viability was observed in cells treated with the extract (almost +30% viability). Under these very low concentration conditions, the extract was not only not cytotoxic but could actually be inducing cancer cell proliferation or protecting them, perhaps by activating cellular metabolic pathways at low doses. Statistical analysis (indicated by the letters on each bar in Figure 4) showed that treatments at 4000 *μ*g/mL were significantly different from those at intermediate concentrations (500–2000 *μ*g/mL, letter b) and at the lowest concentration (125 *μ*g/mL, letter c), demonstrating a clear differential response between treatments. This phenomenon suggests that while fucoidans could exert significant antiproliferative activity at therapeutic levels, subtherapeutic or low concentrations might inadvertently stimulate cell growth. Such behavior has important implications for food and nutraceutical applications, where fucoidan‐containing products may be consumed at variable doses. Careful consideration of effective concentrations and formulation strategies is therefore critical to ensure that the beneficial bioactive properties are harnessed without promoting unintended cellular proliferation. Our results about antitumor activity are consistent with previous research on fucoidan, in which its ability to induce apoptosis, inhibit cell proliferation, and modulate signaling pathways such as PI3K/Akt or MAPK in cancer cell lines, including the HeLa line, has been found [16]. The action observed at high concentrations may be related to the induction of proapoptotic mechanisms or direct damage to the cell membrane, while the response at low concentrations could be associated with the activation of antioxidant or immunomodulatory mechanisms. Overall, the data obtained support the extract′s potential as an antiproliferative agent, but also indicate the need to study its effects at a wider range of concentrations and treatment durations and validate them through complementary alternatives such as flow cytometry or specific apoptosis staining. As observed in Figure 5, cell viability decreases as the concentration of fucoidan increases, indicating a dose‐dependent effect. Fitting the data to an exponentially decreasing curve reveals a good correlation (*R* ^2^ = 0.9037), validating the observed trend. From the generated curve, an IC_50_ of 1277.79 *μ*g/mL is obtained, a value that indicates the concentration of the extract necessary to reduce the viability of HeLa cells by 50%.

The results obtained in this study contrast notably with those reported by Palanisamy et al. [11], who found that fucoidans isolated from *S. polycystum* exhibited a cytotoxicity of 90.4*%* ± 0.25*%* on the MCF‐7 cell line at a concentration of 150 *μ*g/mL, with an estimated IC_50_ of 50 *μ*g/mL. The difference in results could be a consequence of the different extraction and purification techniques, which would result in obtaining fucoidans with different chemical compositions and biological properties. The progressive decrease in viability with increasing concentration, and the IC_50_ value obtained, does support the hypothesis that may be activated by apoptotic pathways. Mechanism‐specific assays to characterize these mechanisms were not included in this study. Interestingly, the slight stimulation of cell viability at low concentrations (125 *μ*g/mL) may speak to a hormetic effect, whereby bioactive compounds elicit opposite responses depending upon dosage. This behavior has been described for several natural extracts and could be related to the provision of transient elicitation of cell defense mechanism activity or mild oxidative stress [10, 17]. Similarly, Bobiński et al. [18] evaluated the activity of fucoidans extracted from brown algae in uterine sarcoma (MES‐SA and ESS‐1) and carcinosarcoma (SK‐UT‐1 and SK‐UT‐1B) cell lines, as well as their toxicity in human fibroblasts (HSF). The results showed that fucoidans significantly reduced cell viability in SK‐UT‐1, SK‐UT‐1B, and ESS‐1, but not in MES‐SA, and did not substantially affect the proliferation of normal HSF cells. On the other hand, fucoidans induced apoptosis in all the lines evaluated, reaching the highest effect in SK‐UT‐1 (79.88%) and the lowest in SK‐UT‐1B (4%). Furthermore, it also caused cell cycle alterations, inducing G0/G1 and sub‐G1 arrest in most cell lines, except in MES‐SA, in which the effect was limited. Finally, fucoidans exhibited very low toxicity in human fibroblasts, demonstrating a promising safety profile. Thus, together, these results show that fucoidan has both cytotoxic and cytostatic activity, which positions it as a promising agent for the treatment of uterine sarcomas and carcinosarcomas, also considering the need to carry out new studies in murine models and clinical trials in patients. Several scientific studies have concluded that fucoidans do not exert negative effects on normal cells. Both the studies carried out by Bobiński et al. [18], Niyonizigiye et al. [19], and the work carried out by Tsou et al. [12] support this statement. These investigations have evaluated the effects of fucoidan on various normal cell lines and have found that there is no significant reduction in cell viability or proliferation.

On the other hand, it has been reported that the biological activity of fucoidans from brown algae depends on their source, purity, and degree of sulfation, and the observed biological properties may be closely related to the structural characteristics of these fucoidans. In our study, we found the degree of sulfation for *S. natans* to be 13.2% and 15.6% for *S. fluitans*. These values fall into the range that is well known to exhibit strong biological effects. Sulfation is one of the major structural characteristics involved in fucoidans′ bioactivity to exert their antioxidant and antitumor effects, because the content of this group and the interaction of these polysaccharides with the cellular receptors will also increase, and this will have an impact on the modulation of the signaling pathways. The Mws of these fucoidans were also estimated from the intrinsic viscosity measurements using the Mark–Houwink equation; they were found to be 26.2 kDa for *S. natans* and 18.7 kDa for *S. fluitans*. Fucoidans with reduced Mw have been reported to be able to exhibit enhanced uptake by the cells and increased cytotoxicity in different in vitro model systems of cancer. Consequently, it is conceivable that the moderate sulfation and the relatively low Mw of these extracts may act as a combination adding up to a synergistic effect that may be able to contribute to the extremely strong antiproliferative effects found at higher concentrations in HeLa cells [10].

Although understanding the exact mechanism by which fucoidans exert their antitumor action can be challenging due to the inherent variability of these compounds and differences in the cell lines used in various studies, the scientific literature suggests that their effects could be mediated by multiple factors. These include the induction of apoptosis, the inhibition of angiogenesis, and the modulation of the immune system [19, 20].

## 4. Conclusions

The results obtained in this study demonstrate the therapeutic potential of fucoidans, particularly their ability to reduce cell viability in cancer cells, such as the HeLa cell line, without significantly affecting the proliferation of normal cells. This behavior suggests potential selective cytotoxicity, which represents an important advantage for their development as an antitumor agent.

A limitation of this study is that the antiproliferative effect was evaluated only in the HeLa cervical cell line, and mechanistic analyses such as apoptotic induction, cell cycle arrest, or molecular marker evaluation were not performed. These aspects will be addressed in future studies using multiple cancer and normal cell lines, along with complementary mechanistic assays, to better understand the pathway underlying the observed effects. It is important to consider that the concentration of fucoidan used in this study (4000 *μ*g/mL) is high and likely difficult to achieve under human physiological conditions due to its limited bioavailability, rapid metabolism, and excretion. These limitations must be taken into account when extrapolating in vitro results to clinical applications and underscore the need to develop more efficient formulation and administration strategies.

Despite the growing interest in fucoidans, a deeper understanding of their mechanisms of action at the cellular and molecular levels is still required, as well as preclinical studies in animal models and human clinical trials that allow a more precise evaluation of their safety and efficacy.

Finally, this work not only highlights the potential therapeutic value of fucoidans in oncology but also opens the possibility of biotechnologically exploiting the massive flooding of *Sargassum*, thus contributing to a sustainable solution to a growing environmental problem.

## Conflicts of Interest

The authors declare no conflicts of interest.

## Funding

No funding was received for this manuscript.

## Data Availability

The datasets generated and analyzed during the current study are available from the corresponding author upon reasonable request.
